# Expression and functional regulation of stemness gene Lgr5 in esophageal squamous cell carcinoma

**DOI:** 10.18632/oncotarget.15624

**Published:** 2017-02-22

**Authors:** Zhuan Lv, Jane J. Yu, Wei-Jie Zhang, Li Xiong, Feng Wang, Li-Feng Li, Xue-Liang Zhou, Xin-Ya Gao, Xian-Fei Ding, Li Han, Ya-Fei Cai, Wang Ma, Liu-Xing Wang

**Affiliations:** ^1^ Department of Oncology, First Affiliated Hospital of Zhengzhou University, Zhengzhou, Henan, China; ^2^ University of Cincinnati College of Medicine, Department of Internal Medicine, Division of Pulmonary, Critical Care and Sleep Medicine, Cincinnati, OH, USA; ^3^ Department of General Surgery, The Second Xiang Ya Hospital of Central South University, Hunan, China

**Keywords:** Lgr5, esophageal squamous cell carcinoma, cancer stem cells, spheroid body cells, small interfering RNA

## Abstract

Cancer stem cells (CSCs) are defined as a rare subpopulation of undifferentiated cells with biological characteristics that include the capacity for self-renewal, differentiation into various lineages, and tumor initiation. To explore the mechanism of CSCs in esophageal squamous cell carcinoma (ESCC), we focused on Leucine-rich repeat containing G protein-coupled receptor 5 (Lgr5), a target gene of the Wnt signaling pathway, which has been identified as a marker of intestinal stem cells and shown to be overexpressed in several human malignancies. Lgr5 expression was significantly correlated with lymph node metastasis, increased depth of invasion, increased tumor size, advanced differentiation, higher AJCC stage and poorer survival. Silencing of Lgr5 expression in the ESCC cell line KYSE450 by small interfering RNA (siRNA) strongly inhibited cell proliferation, migration and invasion ability, the expression of CSCs-related genes and Wnt/β-catenin signaling. In addition, Lgr5 was highly expressed in ESCC spheroid body cells, which were identified by high expression of CSCs-related genes, and high tumorigenicity *in vivo*. Taken together, these results demonstrate that Lgr5 activation of Wnt/β-catenin signaling is a potential mechanism to promote the progression of ESCC and ESCC stem cell renewal, and Lgr5 may be used as a molecular target for the development of treatments for ESCC.

## INTRODUCTION

Esophageal carcinoma (EC) is the eighth most common cancer and the sixth leading cause of cancer death around the world. Esophageal squamous cell carcinoma (ESCC) is the main histopathological subtype of EC in East Asians, with a 5-year survival rate < 30% [1–3]. Although there are advanced therapeutic treatments for ESCC such as chemotherapy, radiotherapy, surgery and target therapy, many ESCC cases progress or recur, and prognoses is poor [4]. Emerging evidence indicates that cancer stem cells (CSCs) contribute to tumor maintenance, tumor progression, therapy resistance, and distant metastasis [5–6]. CSCs are defined as a rare subpopulation of undifferentiated cells with biological characteristics that include the capacity for self-renewal, differentiation into various lineages, and tumor initiation [7]. A critical knowledge gap in CSCs research includes methods to identify and isolate CSCs. Mounting evidence demonstrates that CSCs can be enriched and maintained using a spheroid body formation assay in serum-free medium at low adherence, and isolated using fluorescence-activated cell sorting (FACS) according to CSCs-specific cell surface markers [8, 9]. CSCs express distinct surface markers, allowing for reproducible and differential purification. Several stem cell markers, such as SOX2, OCT4, NANOG, KLF4 and ALDH1A1, have been successfully used to identify CSCs in tumor tissues [10, 11].

Lgr5 is a member of the G-protein-coupled receptor (GPCR) family of proteins, and is a regulated target of Wnt signaling. Lgr5 contains a large extracellular domain with 17 leucine-rich repeats and a seven-transmembrane domain typical of the rhodopsin family of GPCRs [12–14]. Barker et al. discovered that Lgr5 expression is restricted to the crypt of the small intestine and colon and that single sorted Lgr5+ crypt base columnar cells can also initiate crypt-villus organoids. Therefore, Lgr5 is a marker of stem cells in the small intestine and colon [15–17].

In recent years, studies have revealed that Lgr5 is overexpressed in various types of tumors, including colorectal cancer, hepatocellular carcinoma, ovarian cancer, glioma, basal cell carcinoma and gastric carcinoma [18–20]. The Wnt/β-catenin signaling pathway plays a critical role in the regulation of stem and cancer stem cells, and abnormally activated Wnt/β-catenin pathway is usually associated with tumorigenesis, including ESCC [21, 22]. Lgr5 acts as receptor for the Wnt/β-catenin signaling agonist R-spondin [23, 24]. RSPO2 is a member of the R-spondin family and a specific ligand of Lgr5. RSPO2 might also enrich CSCs by regulating Lgr5 expression in cancer cells [25]. Recently, it has been shown that RSPO2-Lgr5 signaling has been shown to potentiate Wnt/β-catenin signaling in other tissues, particularly in colorectal carcinoma [26].

To date, little research has been conducted on Lgr5 in ESCC, and the mechanism by which Lgr5 modulates ESCC and the stemness of ESCC remain to be elucidated. In this present study, we investigated the role of Lgr5 in ESCC and ESCC spheroid body cells, which were identified as a small subset of stem-like cancer cells. Our findings demonstrated that Lgr5 was progressively expressed in esophageal carcinogenesis, and Lgr5 expression promoted cell proliferation, cell migration and invasion, the expression of CSCs-related genes, and activated the Wnt/β-catenin signaling pathway. These data demonstrate that Lgr5 may be used as a molecular target for the development of treatments for ESCC.

## RESULTS

### Lgr5 is overexpressed in ESCC and expression is negatively correlated with prognosis

**Figure 1:**
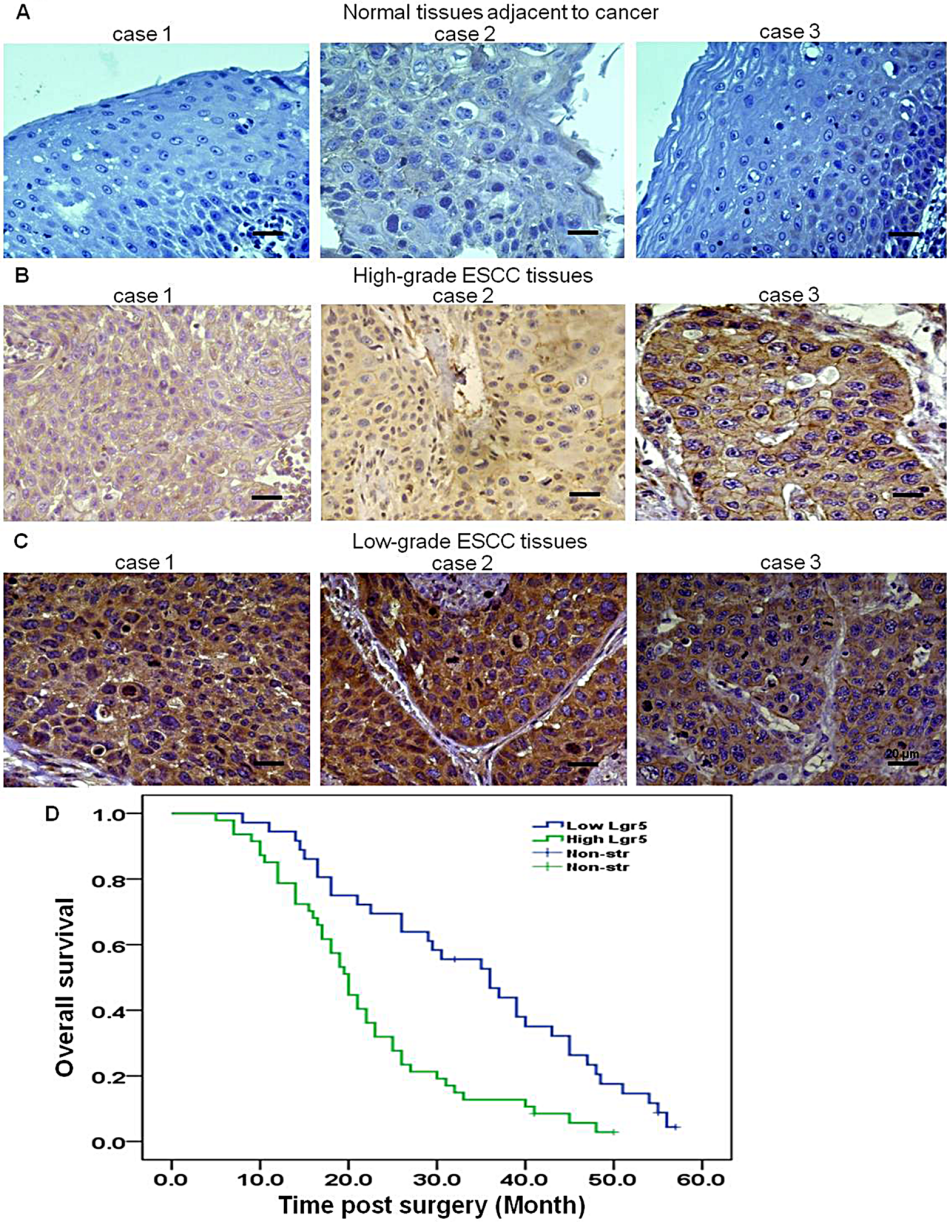
Lgr5 is overexpressed in ESCC, and expression is negatively correlated with prognosis Representative images of immunohistochemical staining of Lgr5 in (**A**) three different adjacent normal esophageal squamous epithelial tissues (*n* = 98, ×400, scale bar, 20 μm), (**B**) three different high-grade ESCC tissues (*n* = 93, ×400, scale bar, 20 μm) and (**C**) three different low-grade ESCC tissues (*n* = 95, ×400, scale bar, 20 μm). (**D**) Kaplan-Meier survival analysis indicated a correlation between high expression of Lgr5 and poorer overall survival in ESCC patients.

We examined the expression of Lgr5 in ESCC and normal esophageal squamous epithelial tissues by immunohistochemistry. Lgr5 protein was positively expressed in 67% (188/280) of the ESCC, and 35% (52/150) of normal esophageal squamous epithelial tissues samples. Expression of Lgr5 protein was found predominantly in the cytoplasm and membrane of cancer cells, with some nucleus staining (Table 1 and Figure 1A–1C). A significant difference in Lgr5 expression was found between ESCC and normal esophageal squamous epithelial tissues (*χ^2^* = 19.285, *P* < 0.0001). We also compared Lgr5 expression and clinicopathological characteristics in ESCC patients by immunohistochemistry. Lgr5 expression was significantly correlated with lymph node metastasis (*χ^2^* = 8.351, *P* = 0.040), increased depth of invasion (*χ^2^* = 15.95, *P* < 0.0001), increased tumor size *(χ^2^* = 8.819, *P* = 0.012), advanced differentiation (*χ^2^* = 14.249, *P* = 0.001) and higher AJCC stage (*χ^2^* = 4.99, *P* = 0.025) (Table 2). No significant association was found between Lgr5 expression and age or gender (all *P* > 0.05). Kaplan-Meier analysis suggested that prognosis was poor for patients with high Lgr5 expression (Figure 1D).

**Table 1:** The expression of Lgr5 in esophageal squamous cell carcinoma tissues and normal esophageal squamous epithelial tissues

	*N*	Lgr5 protein expression/n		χ^2^	*P*
		Negative (−)	Positive (+)		
Esophageal squamous cell carcinoma tissues	280	92	188	19.285	< 0.0001
Normal tissues adjacent to cancer	150	98	52		

**Table 2:** Lgr5 expression and clinicopathological characteristics in ESCC patients

Clinicopathological Characteristics	*N*	Lgr5 protein expression/n		χ^2^	*P*
		Negative (−)	Positive (+)		
Age/year					
≥ 61	132	40	92	0.738	0.390
< 61	148	52	96		
Gender					
Male	181	63	118	0.882	0.348
Female	99	29	70		
Lymph node metastasis					
Yes	156	40	116	8.351	0.040
No	124	52	72		
Depth of invasion					
T1	73	34	39	15.95	< 0.0001
T2	99	37	62		
T3	108	21	87		
Tumor size (cm)					
d < 3	62	30	32	8.819	0.012
3 cm ≤ d < 5	70	21	49		
≥ 5	148	41	107		
Differentiation					
Well	48	22	26	14.249	0.001
Moderately	112	45	67		
Poorly	120	25	95		
AJCC stage					
I+II	105	43	62	4.99	0.025
III+IV	175	49	126		

### ESCC KYSE450 cells form spheroid bodies

**Figure 2:**
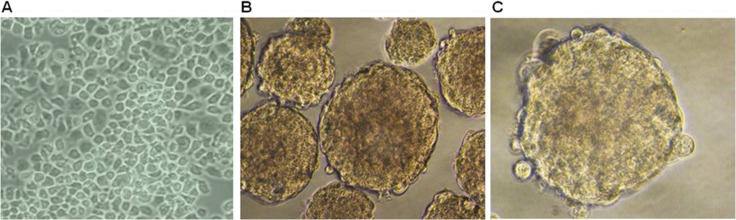
Spheroid formation is evident in ESCC KYSE 450 cells (**A**) Morphology of cells grown in RMPI 1640 medium supplemented with 10% FBS (×200). (**B**, **C**) Cells cultured in stem cell specific culture media. Cell morphology shows formation of spheroids (×400).

Mounting evidence suggests that CSCs properties could be propagated *in vitro* as non-adherent spheres under serum-free culture conditions [27, 28]. Using ultra low attachment surface plates and serum-free culture conditions supplemented with B27, bFGF, EGF and heparin, ESCC KYSE450 cells grew as non-adherent, three-dimensional spheroid bodies after seven days (Figure 2). These spheroid body cells could be dissociated into single cells, which indicated they have the capacity of self-renewing.

### Lgr5, CSCs-related genes and RSPO2 are overexpressed in ESCC KYSE450 spheroid body cells

**Figure 3:**
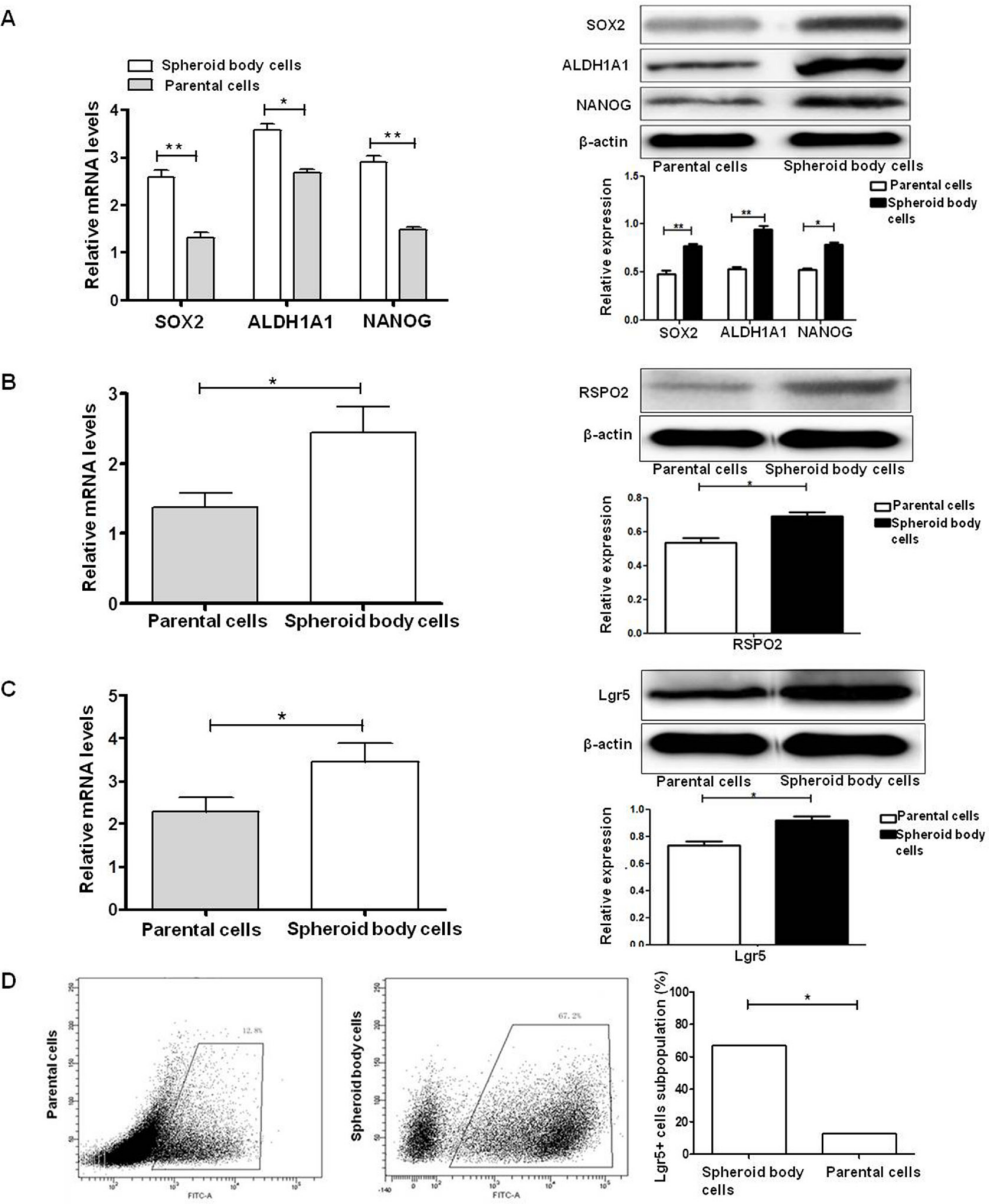
Lgr5, CSC-related genes, and RSPO2 are overexpressed in ESCC KYSE450 spheroid body cells (**A**) mRNA and protein levels of SOX2, ALDH1A1, and NANOG are up-regulated in spheroid body cells *P < 0.05, **P < 0.01, vs the parental cells group). (**B**) qRT-PCR and western blot analysis demonstrated elevated mRNA and protein levels of RSPO2 in KYSE450 spheroid body cells compared with parental cells (*P < 0.05). (**C**) qRT-PCR and western blot analysis demonstrated elevated mRNA and protein levels of Lgr5 in KYSE450 spheroid body cells compared with parental cells (*P < 0.05). (**D**) Flow cytometric analysis of the Lgr5^+^ cell subpopulation in KYSE450 spheroid body cells (67.2%) and parental cells (12.8%).

A growing body of evidence demonstrates that SOX2, ALDH1A1 and NANOG are important stemness genes for many CSCs and play crucial roles in self-renewing, differentiation and tumorigenicity of CSCs [29, 30]. RSPO2 is a member of the R-spondin family proteins that are secreted agonists of the canonical Wnt pathway, which act through binding with LGRs. qRT-PCR and western blot analyses were performed on spheroid body cells and parental cells. We found that ESCC KYSE450 spheroid body cells overexpressed, ALDH1A1, NANOG and the specific ligand of Lgr5, RSPO2, compared to ESCC KYSE450 parental cells (Figure 3A, 3B). To further examine the expression of Lgr5 in ESCC KYSE450 spheroid body cells, qRT-PCR, western blot and flow cytometric analyses were performed on the spheroid body cells and parental cells (Figure 3C, 3D). The results of qRT-PCR and western blot indicated that the protein and mRNA levels of Lgr5 were elevated in ESCC KYSE450 spheroid body cells, compared with ESCC KYSE450 parental cells. Flow cytometric analysis revealed that KYSE450 spheroid body cells contained a high proportion of Lgr5^+^ cells, while parental cells had a smaller Lgr5^+^ fraction. These results indicate that ESCC KYSE450 spheroid body cells have an increased expression of CSC-related genes. Moreover, in these spheroid body cells, the expression of Lgr5 and its specific ligand, RSPO2, were increased.

### Spheroid body cells display high tumorigenic potential *in vivo*

**Figure 4:**
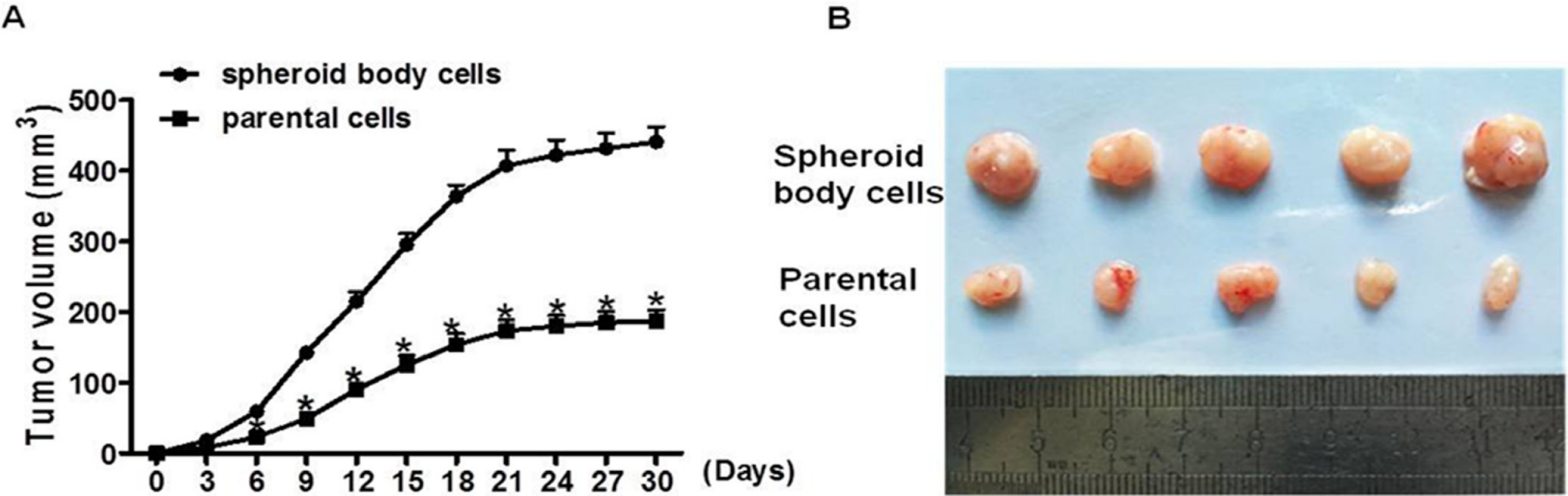
Spheroid body cells display high tumorigenic potential *in vivo* 5 × 10^5^ spheroid body cells or 5 × 10^5^ parental cells were injected subcutaneously into BALB/c nude mice (*n* = 5/group). Xenograft tumors developed 4 weeks post cell injection. Tumor size was measured every three days. Tumor volume was calculated as length ×width ×depth. (**A**) The average of tumor volumes was plotted. (**B**) Xenograft tumors were resected from mice at 4 weeks post cells injection.

The most convincing demonstration of CSCs identity is their enhanced tumor-initiating capacity *in vivo*, which is undoubtedly regarded as the gold standard for evaluating the existence of CSCs [31]. To further identify the stemness of spheroid body cells and examine their tumor initiating capability, 5 × 10^5^ spheroid body cells or 5 × 10^5^ parental cells were injected into BALB/c nude mice. There was a significant difference in tumor-initiating capacity between these groups; tumor volumes and tumor sizes were higher in mice injected with spheroid body cells, compared with parental cells (Figure 4A, 4B). These results suggest that spheroid body cells were significantly more tumorigenic than the parental cells, and the stemness of the spheroid body cells was enhanced.

### Silencing of Lgr5 inhibits the proliferation, migration and invasion of ESCC cells

**Figure 5:**
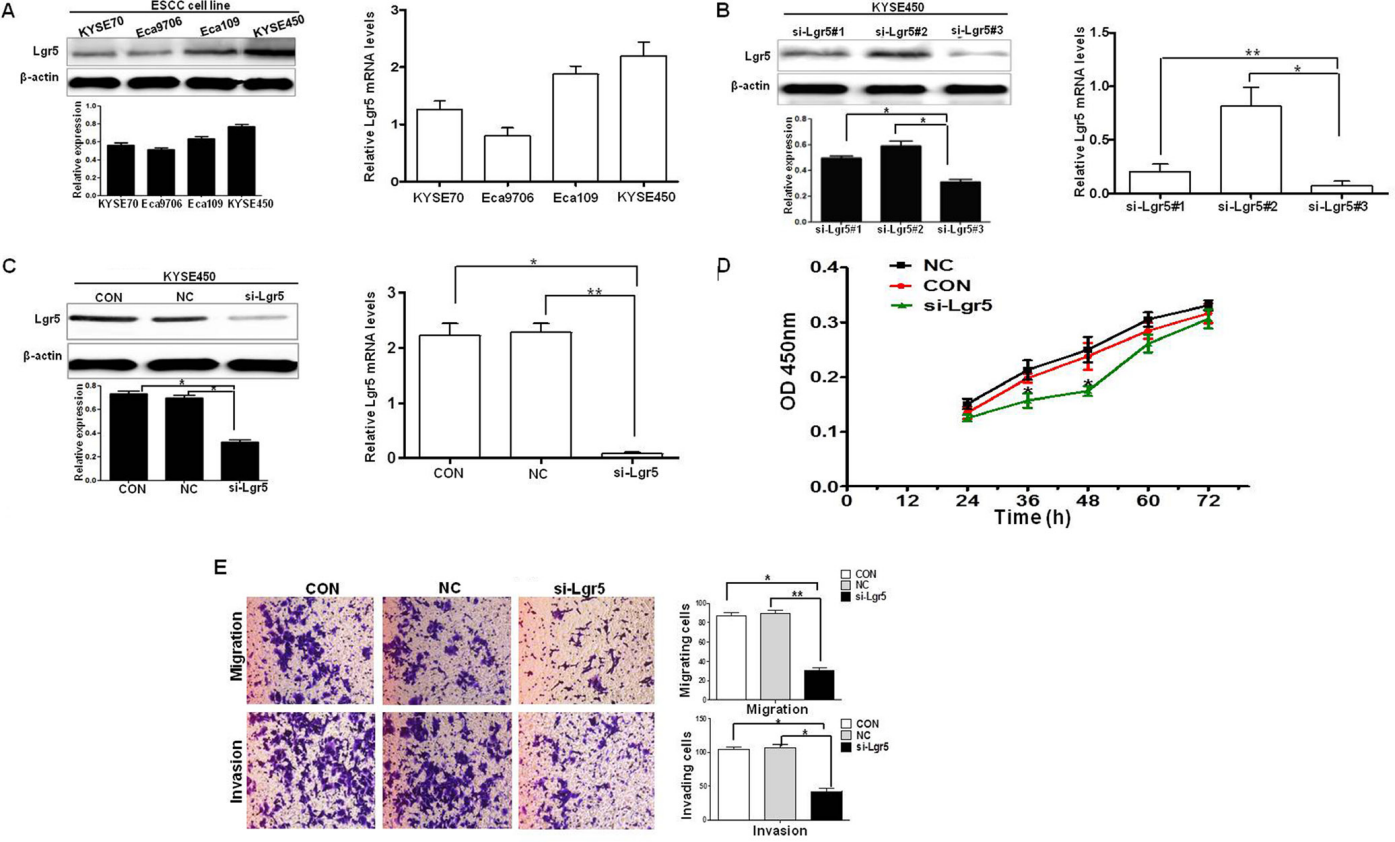
Silencing of Lgr5 inhibits the proliferation, migration and invasion (**A**) Lgr5 expression in ESCC cell lines is shown by western blot and qRT-PCR assays. (**B**) Silencing of Lgr5 expression by siRNA in KYSE450 cells is shown 48 h after transfection by Western Blot and qRT-PCR assays (*P < 0.05, **P < 0.01, si-Lgr5#3 vs si-Lgr5#1, si-Lgr5#2). (**C**) Protein and mRNA expression were compared 48 h after transfection with Lgr5-targeting siRNA against CON group cells and NC group cells (*P < 0.05, **P < 0.01 si-Lgr5 vs CON group, NC group). (**D**) Cell proliferation assay revealed that Lgr5 inhibition reduced cancer cell proliferation in ESCC KYSE450 cells. (**E**) Transwell migration and invasion assays revealed that inhibition of Lgr5 resulted in decreaseed cell proliferation in ESCC KYSE450 cells (*P* = 0.007, *P* = 0.005).

We investigated Lgr5 expression in four esophageal squamous cell carcinoma cell lines (KYSE70, Eca9706, Eca109, and KYSE450) by western blot and qRT-PCR, respectively (Figure 5A). Results showed that the KYSE450 cell line had the highest level of Lgr5 expression. For subsequent siRNA analyses, we generated three Silencer Select siRNA to target different regions of the Lgr5 transcript. The three siRNA efficiently inhibited the expression of Lgr5 in KYSE450 cells (Figure 5B). We used si-Lgr5#3 in the follow-up experiments. In order to assess the efficiency of si-Lgr5 to downregulate expression of Lgr5, western blot and qRT-PCR were performed at 48 hours after siRNA transfection. Both protein and mRNA expression levels of Lgr5 were significantly decreased in the si-Lgr5 group compared with the control group (CON) and negative control group (NC, a non-specific scramble siRNA group) (*P* < 0.05, Figure 5C). We investigated the proliferation of Lgr5 cells at 24, 36, 48, 60, and 72 h after transfection by using the CCK-8 assay. The number of ESCC cells in the si-Lgr5 group was significantly reduced compared with either the NC group or CON group (*P* < 0.05, Figure 5D). Subsequently, transwell migration and invasion assays were used to examine the effects of Lgr5 on the migration and invasiveness of ESCC cells. In the migration assay, si-Lgr5 cells showed reduced migratory ability compared to NC group and CON group cells (si-Lgr5 versus NC group and CON group, 28.32 ± 9.7 versus 83.56 ± 16.32 and 81.28 ± 14.7; *P* < 0.05, Figure 5E). Similarly, in the invasion assay, si-Lgr5 cells showed reduced invasion ability compared to NC group and CON group cells (si-Lgr5 versus NC group and CON group, 31.89 ± 20.53 versus 99.48 ± 18.7 and 95.62 ± 20.31; *P <* 0.05, Figure 5F). These results indicate that siRNA mediated specific down-regulation of Lgr5 and induced strong inhibition of ESCC cell growth, migration and invasion.

### Silencing of Lgr5 reduces the expression of CSCs-related genes

**Figure 6:**
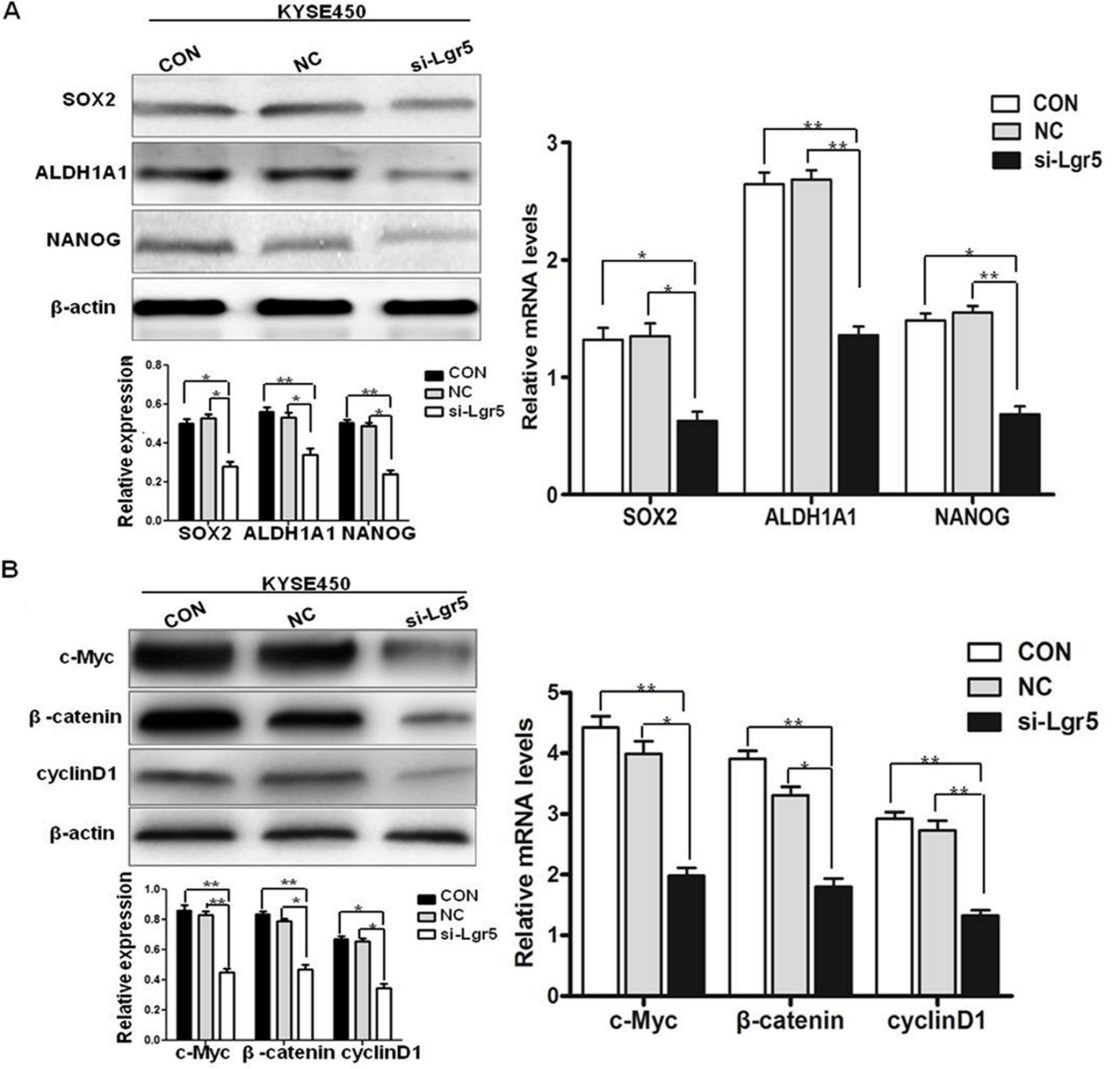
Silencing of Lgr5 enhances the expression of CSCs-related genes and the activity of the Wnt/β-catenin pathway (**A**) Protein and mRNA levels of CSCs-related genes at 48 h after transfection were compared in Lgr5-targeting siRNA against CON group cells and NC group cells (*P < 0.05, **P < 0.01, si-Lgr5 vs CON group, NC group). (**B**) Protein and mRNA levels of β-catenin, c-Myc, and cyclinD1 at 48 h after transfection were compared among Lgr5-targeting siRNA against CON group cells and NC group cells (*P < 0.05, **P < 0.01, si-Lgr5 vs CON group, NC group).

It is well known that CSCs-related genes play an important role in CSCs stemness. As we identified above, the expression of CSCs-related genes SOX2, ALDH1A1 and NANOG were elevated in ESCC spheroid body cells. To further identify the relationship between ESCC cells and CSCs-related genes, we examined the protein and mRNA levels of the CSCs-related genes SOX2, ALDH1A1 and NANOG in KYSE450 cells. Protein and mRNA levels at 48 h after transfection were compared in si-Lgr5 group cells, CON group cells, and NC group cells. The mRNA and protein levels of SOX2, ALDH1A1 and NANOG were reduced in the si-Lgr5 group compared with NC group and CON group, implying that the silencing of Lgr5 in ESCC cells may reduce CSCs properties (Figure 6A).

### Silencing of Lgr5 reduces activity of the Wnt/β-catenin pathway

β-catenin is a crucial signaling molecule, and cyclinD1 and c-Myc are important target genes of the Wnt/β-catenin signaling pathway. Previous studies have shown that Lgr5 could bind with its specific ligand RSPO2 to enhance Wnt/β-catenin signaling activity. Moreover, increasing evidence shows that Lgr5 is involved in Wnt/β-catenin pathway in the regulation of cancer stem cells. We compared the expression of β-catenin, cyclinD1, and c-Myc protein and mRNA to confirm that the oncogenic effects of Lgr5 are mediated through activation of the Wnt/β-catenin pathway. Protein and mRNA levels of β-catenin, cyclinD1, and c-Myc in the si-Lgr5 group were reduced, compared with NC group and CON group (Figure 6B). These results demonstrate that Lgr5 expression is positively associated with the activity and expression of key molecules of the Wnt/β-catenin pathway in ESCC, implicating Lgr5 as an activator of the Wnt/β-catenin signaling pathway.

## DISCUSSION

Cancer stem cells (CSCs) were first identified in patients with acute myeloid leukemia [32], and subsequently discovered in other malignancies including brain, prostate, lung, breast, colon, melanomas and pancreatic cancers [8, 33–35]. CSCs are a small population of cancer cells, with self-renewal and differentiation potential, and play a distinct role in tumor initiation, progression, metastasis, immune evasion and recurrence [7–8, 36]. Previous studies have reported that CSCs could be successfully isolated and identified by three methods: spheroid body formation, fluorescence-activated cell sorting (FACS), and side population (SP) cells. Spheroid body cell culture is used to functionally enrich and identify potential CSCs subpopulations from various types of cancers such as breast cancer, hepatoma and prostate cancer [37, 38]. In the present study, we developed spheroid body cells by cultivating the human ESCC cell line KYSE450 in a defined ultra-low attachment surface culture. ESCC KYSE450 cells could form spheroid body cells, indicating stemness characteristics. We further evaluated the stemness characteristics of ESCC spheroid body cells by evaluating the expression of the CSCs-related genes SOX2, ALDH1A1 and NANOG. SOX2 is a stemness gene of ESCC, is required for ESCC cell growth, and that SOX2 overexpression in esophageal epithelial cells induces migration and transforms growth and tumor formation *in vivo* [39]. Sato et.al showed that ovarian epithelial cancer cells and tumors express high levels of ALDH1A1 compared with normal cells, suggesting ALDH1A1 as a potential biomarker for CSCs [40]. NANOG is overexpressed in ovarian carcinoma, colon carcinoma and ESCC, and enhances proliferation and self-renewal of pluripotent stem cells [41]. The most convincing demonstration of CSCs identity is their enhanced tumor-initiating capacity *in vivo*, which is undoubtedly regarded as the gold standard for evaluating the existence of CSCs [42]. We found that ESCC KYSE450 spheroid body cells overexpress the stemness genes SOX2, ALDH1A1 and NANOG, compared with ESCC KYSE450 parental cells. We also observed that ESCC spheroid body cells are highly tumorigenic in xenograft models, while ESCC parental cells were less tumorigenic. Other studies have shown that Lgr5 is also overexpressed in tumor spheroid cells in colorectal cancer and breast cancer [43, 44], similar to our findings. Both Lgr5 gene and protein expression were enhanced in ESCC spheroid body cells compared with parental cells. These results indicate that Lgr5 positive spheroid body cells might represent a kind of ESCC cancer stem cell, and Lgr5 might be a stemness gene in ESCC.

Studies have demonstrated that Lgr5 is overexpressed in various tumors [18–20]. In these tumors, Lgr5 has been associated with depth of invasion, lymph node metastasis and distant metastasis [45]. Similar to our findings, Lgr5 was overexpressed in ESCC tissues (67%) compared with normal esophageal squamous epithelial tissues (35%). Additionally, we showed a relationship between Lgr5 expression and clinicopathological features in ESCC. Lgr5 expression was significantly correlated with lymph node metastasis, increased depth of invasion, increased tumor size, advanced differentiation, higher AJCC stage and poor survival. Accumulating data has demonstrated that the silencing of Lgr5 influences the functional and molecular outcome of colorectal cancer cells, breast cancer cells, cervical cancer and ovarian cancer [25, 43, 46–47]; for example, previous reports indicated that knocking down endogenous Lgr5 in cultured colorectal cancer cell lines reduced their proliferation, migration, invasion, growth rates and colony formation capability. In order to investigate the biological role of Lgr5 in ESCC cells, we verified that silencing of Lgr5 could inhibit cell proliferation, migration and invasion, and the levels of CSC-related genes, which act together to promote tumorigenesis and accelerate the progression of ESCC. Taken together, these findings suggest that Lgr5 may be a potential target for patients with aggressive and metastatic tumors, as well as a prognostic marker for ESCC.

Recently, accumulating evidence supports the hypothesis that the Wnt/β-catenin pathway is correlated with function and renewal of cancer stem cells [48]. Studies have demonstrated that Lgr5 promotes tumorigenesis, accelerates cell proliferation, and activates the Wnt/β-catenin pathway [49]. Expression of β-catenin, cyclinD1 and c-Myc was significantly decreased in cervical cancer cells depleted with Lgr5 compared with that in control cells, indicating that Lgr5 expression is positively associated with the expression and activity of Wnt/β-catenin pathway mediators in cervical cancer cells. Moreover, Lgr5-promoted proliferation and tumor progression of cervical cancer cells *in vivo* is possibly mediated by Wnt/β-catenin pathway. Lu et al. reported that Lgr5 promotes growth and invasion, and maintains the stemness of breast cancer cells via Wnt/β-catenin pathway [43]. In this study, we show that silencing of Lgr5 reduces the expression of β-catenin and its effector genes cyclinD1 and c-Myc in ESCC cells. We also show that Lgr5 activates the β-catenin signaling pathway, leading to ESCC progression.

Lgr5 binds to its ligands, RSPO members, to activate Wnt/β-catenin pathway [21, 22]. Ilmer et al. showed that RSPO2-enhanced canonical Wnt signaling contributes to cancer stemness in pancreatic cancer [50]. Lgr5 and RSPO2 are also associated with tumor aggressiveness, lymph node metastases, and Wnt/β-catenin activation in human papillary thyroid cancer [51]. higher percentage of Lgr5+ CSCs and Overexpression of RSPO2 and Lgr5 was identified in spheroid cells. Studies also indicated that RSPO2 enriches colorectal cancer stem cells by increasing the expression of Lgr5 [26]. In agreement with these studies, we found that the expression of Lgr5 and RSPO2 was significantly elevated in spheroid cells compared to adherent cells, and RSPO2 might have the same effect on enriching and maintaining ESCC cancer stemness.

Taken together, our results demonstrate that Lgr5 expression is significantly correlated with several clinicopathological factors, Lgr5 is enhanced in both ESCC tissues and ESCC spheroid body cells, and patients with high Lgr5 expression have poor prognoses. Lgr5 overexpression is a potential biological marker for tumorigenesis, progression, migration and invasion in ESCC, and Lgr5 might be a regulatory mechanism of stemness in ESCC. Lgr5 activates the β-catenin signaling pathway, including the downstream target genes cyclin D1 and c-Myc, leading to progression of ESCC. Lgr5 may play a key role in maintaining ESCC stem-like cells through Wnt/β-catenin signaling. Lgr5 and its ligand, RSPO2, might have the same effect on enriching and maintaining ESCC cancer stem cells. Thus, Lgr5 has the potential to be an important cell surface marker of ESCC stem cells, and it may be used as a molecular target for the development of treatments for ESCC. As the Wnt-Lgr5-RSPO2 axis plays a key role in CSCs, we plan to further dissect the roles of Lgr5 and RSPO2 in maintaining stemness through activation of Wnt/β-catenin signaling in ESCC.

## MATERIALS AND METHODS

### Patients and tissue specimens

Enrollment consisted of 280 patients with ESCC who underwent surgical resection with curative intent from 2012 to 2014 at the First Affiliated Hospital of Zhengzhou University, Zhengzhou, China. An additional 150 normal esophageal squamous epithelial tissue samples adjoining cancer tissues were included as controls. Of the 280 patients with ESCC, 105 were classified as stage I+II and 175 as stage III+IV. The patient ages were between 40 and 82 (median 61) years old; 181 were men and 99 were women. According to pathological differentiation, 48 patient samples were classified as well differentiated, 112 samples as moderately differentiated, and 120 patient samples were poorly differentiated. Lymph node metastasis was noted in 156 ESCC cases. The study protocol was approved by the Ethical Committee for Human Cancer Research of Zhengzhou University.

### Immunohistochemical staining and scoring

Immunohistochemical staining was performed on 3-μm, formalin-fixed, paraffin-embedded sections, as described previously [52]. Briefly, endogenous peroxidase activity was blocked using 3% H_2_O_2_ for 20 min. Sections were incubated with normal goat serum for 30 min to block nonspecific antibody binding sites. Sections were incubated with mouse anti-human Lgr5 antibody (dilution at 1:50, Abcam, USA) overnight at 4°C, and then incubated with a biotin-free HRP-labeled polymer of the EnVision Plus detection system (Dako, Tokyo, Japan) Positive reactions were visualized with DAB solution, followed by counterstaining with hematoxylin. Lgr5 staining was scored on the basis of the percentage of positively stained cells and the staining intensity according to previously published reports [52]. The percent positivity was scored as: 0 for 0%; 1 for 1–25%; 2 for 26–50%; 3 for 51–75%; and 4 for > 75%. The staining intensity was scored as: 0, no staining; 1, weakly stained; 2, moderately stained; and 3, strongly stained. Both percent positivity of cells and staining intensity were scored in a double blinded manner. The staining of Lgr5 was assessed as: -, a final staining score < 3; +, a final staining score of 3; ++, a final staining score of 4; and +++, a final staining score of ≥ 5. The final staining score ≥ 4 was classified as positive expression (Lgr5 high) and < 4 as negative expression (Lgr5 low) in ESCC patients.

### Culture of parental and spheroid body cells

Esophageal cancer cell line KYSE450 was preserved in our laboratory and maintained in RMPI 1640 (Hyclone, USA) supplemented with 10% fetal bovine serum (Hyclone, USA), 100 IU/ml of penicillin, and 100 μg/ml of streptomycin at 37°C in a humidified 5% CO_2_ incubator (Thermo, USA). For spheroid cells, we used serum-free DMEM/F12 medium (Invitrogen, USA) supplemented with B27 (1:50, GIBCO, USA), 20 ng/mL bFGF (Pepro Tech, USA), 20 ng/mL EGF (Pepro Tech, USA), 4 μg/mL heparin (Sigma, USA), penicillin 100 IU/mL and streptomycin 100 μg/mL in Ultra Low Attachment Culture Flask (Coring, USA). After seven days, cell morphology was examined under light microscopy, and spheres were collected for experimental analysis or cultured to generate spheres of the next generation.

### Small interfering RNA silencing

Silencing of endogenous Lgr5 was carried out in human esophageal cancer-derived cell lines KYSE450, KYSE70, Eca109 and Eca9706. Knockdown of the endogenous Lgr5 was accomplished using siRNA oligonucleotides for Lgr5 and a negative control (NC, a non-specific scramble siRNA group), purchased from GenePharma Company (Shanghai, China). Three independent oligonucleotides were used for Lgr5 siRNA. The si-Lgr5#1 sequence was 5 ′-GCAGAAUAAUCAGCUAAGATT-3′. The si-Lgr5#2 sequence was 5′GCUCCAGCAUCACUUAUGATT-3′. The si-Lgr5#3 sequence was 5′-GGACGACCUUCA UAAGAAATT-3′. Transfection was performed in 60%-70% confluent cells using Lipofectamine 2000 in Opti-MEM ^®^I (GIBCO, USA) according to the manufacturer's protocol. Briefly, 100 pmol of siRNA and 10 μl of Lipofectamine 2000 were mixed in 500 μl of Opti-MEM ^®^I medium. After 20 min of incubation, the mixture was added to the cells. Forty-eight hours after transfection, cells were analyzed for all experiments.

### RNA extraction and qRT-PCR

Total RNA was extracted from pools of parental esophageal cancer-derived cell lines, spheroid body cells, and cells after si-Lgr5 transfection was quantified, using TRIzol reagent (Invitrogen, USA). cDNA was synthesized using the PrimeScripts RT reagent kit (Takara, Japan). Quantitative real-time polymerase chain reaction (qRT-PCR) was performed using Fast Start Essential DNA Green Master (Roche, USA) and assessed by Agilent Mx3005P. Lgr5 primer sequences were 5′-AAGCAGAGATGCTGCTCCAC-3′(forward) and 5′-GTGAAGACGCTGAGGTTGGA-3′(reverse). β-actin primer sequences were 5 '-TTG GTATCGTGGAAGGACTCA-3' (forward) and 5'-TGTCAT CATATTTGGCAGGTT-3' (reverse). Other primer sequences and additional PCR conditions are available upon request. Fold induction values were calculated using the 2^−ΔΔCt^ method. All experiments were performed in triplicate and repeated at least three times in separate experiments.

### Western blot analysis

Parental cells, spheroid body cells, and cells after si-Lgr5 transfection were lysed as described previously [53]. Lysates were solubilized in Laemmli sample buffer by boiling and then each protein sample was resolved by SDS-polyacrylamide gel electrophoresis with subsequent transfer onto a polyvinylidene difluoride (PVDF) membrane. All membranes were immunoblotted overnight at 4°C with Lgr5 polyclonal antibody (Abcam, USA), ALDH1A1, NANOG, SOX2, E-cadherin, β-catenin, c-Myc, cyclinD1 RSPO2 or β-actin antibody (all from Pepro Tech, USA). Signals were detected by incubation with secondary antibodies labeled with the ECL Detection System.

### Flow cytometry

Parental cells and spheroid body cells were digested with trypsin, transferred to a 5-mL tube, and washed twice with PBS. Subsequently, cell suspensions were incubated with 1:50 anti-Lgr5 Ab (Abcam, USA) in the dark at 4°C for 30 minutes. After the cells were washed 3 times with PBS, FITC Donkey anti-rabbit IgG (BioLegend, USA) was added to the resuspended cells and incubated at 4°C for 30 minutes. The cells were then washed twice in PBS and resuspended in 300 μl PBS for flow cytometric analysis within 1 h.

### Cell proliferation, migration and invasion assays

Transfected cells were seeded at a density of 3000 cells per well in 96-well plates. Medium in each well was changed daily. Viable cell numbers were measured 24, 36, 48, 60, and 72 h after transfection using the Cell Counting Kit-8 (CCK8) (Dojin Laboratories, Kumamoto, Japan), according to the manufacturer's instructions. The absorbance value of each well was measured with a microplate reader set at 450nm. Biocoat Matrigel-coated invasion chambers (BD Biosciences, CA, USA) were used to examine migration and invasion of transfected cells. For the migration assay, 5 × 10^4^ cells were seeded in the upper chamber. For the invasion assay, 5 × 10^4^ cells were seeded in the upper chamber of a transwell insert coated with Matrigel. 500 μl serum-free medium with 10% FBS was added in the bottom chamber. After 15 hours of incubation at 37°C, non-invading cells were removed with a cotton swab, and the invading cells were stained with 1% toluidine blue and counted under an inverted microscope.

### Xenograft experiments

The animal protocol was approved by the Institutional Ethics Review Board of Zhengzhou University and the animals were cared for and used in accordance with established guidelines. Either 5×10^5^ spheroid body cells or 5×10^5^ parental cells were washed with PBS and then suspended in serum-free RPMI/Matrigel mixture (1:1 volume) followed by injecting s.c. into 5-week-old female BALB/c nude mice (5 mice per group). The size and incidence of subcutaneous tumors were recorded.

### Statistical analysis

Statistical analyses were performed using SPSS version 17.0. Correlations between clini-pathologic parameters and Lgr5 expression were analyzed by the *χ^2^* test. Quantitative data were expressed as mean ± standard deviation, unless stated otherwise. Kaplan-Meier plots and log-rank tests were used for survival analysis. *P* < 0.05 was considered significant.
